# Patterns, Predictors, and Mechanisms of Injury in Libyan CrossFit Athletes: A Cross-Sectional Analysis

**DOI:** 10.3390/ijerph23030286

**Published:** 2026-02-25

**Authors:** Sami Elmahgoub, Wesam A. Debes, Adel El Taguri, Mohamed I. Mabrouk, Csaba Melczer, Ahmed B. Bekheet, Ibrahim Affan, Pongrác Ács

**Affiliations:** 1 Department of Physiotherapy, Faculty of Allied Medical Sciences, Applied Science Private University, Amman 11931, Jordan; w_aldebes@asu.edu.jo (W.A.D.); m_mabrouk@asu.edu.jo (M.I.M.); 2 Community Medicine Department, Faculty of Medicine, University of Tripoli, Tripoli 13932, Libya; tajoury@doctor.com; 3 Institute of Physiotherapy and Sports Science, Faculty of Health Sciences, University of Pécs, H-7621 Pécs, Hungary; csaba.melczer@etk.pte.hu; 4 Department of Physical Therapy for Orthopedic Disorders, El Sahel Teaching Hospital, Cairo 11697, Egypt; 5 Physiotherapy Department, Faculty of Physical Therapy, University of Tripoli, Tripoli 13275, Libya; 6 Physical Activity Research Group, János Szentágothai Research Center, University of Pécs, H-7624 Pécs, Hungary; pongrac.acs@etk.pte.hu

**Keywords:** CrossFit, injury prevalence, Libya, musculoskeletal injuries, shoulder

## Abstract

**Highlights:**

**Public health relevance—How does this work relate to a public health issue?**

- This study addresses the public health issue of sports-related musculoskeletal injuries, which contribute to pain, disability, and healthcare costs, and can lead to long-term physical inactivity.
- It investigates injury patterns in a novel and understudied population (Libyan CrossFit athletes), highlighting how gaps in sports infrastructure (e.g., coaching standards) can become a public health concern.

**Public health significance—Why is this work of significance to public health?**

- It provides the first epidemiological data on CrossFit injuries from North Africa, filling a critical geographical gap in global sports injury surveillance.
- Contrary to common assumptions, longer training duration was associated with higher injury risk, suggesting that current injury prevention efforts may be inadequate for experienced athletes. This finding underscores the need for continuous, quality coaching throughout an athlete’s participation, not just during the beginner phase.

**Public health implications—What are the key implications or messages for practitioners, policy makers and/or researchers in public health?**

- The finding that longer training duration predicted injury challenges common assumptions and highlights a gap in secondary prevention, emphasizing the need for ongoing quality coaching even among experienced athletes.
- (1) For practitioners/coaches: Mandates a shift in practice towards mandatory foundational training and ongoing technique coaching to prevent “sudden movement” injuries, rather than focusing solely on intensity. (2) For policy/sports bodies: Highlights the urgent need to develop and enforce local standards for coach certification and gym affiliation in developing fitness markets to safeguard participants and ensure the sustainable growth of community-based exercise.

**Abstract:**

Background: CrossFit is a high-intensity training modality experiencing global growth, but its injury risk profile remains debated. Existing epidemiological studies show a significant geographical bias, with a complete lack of data from North Africa, including Libya. To the best of our knowledge, this study provides the first epidemiological data on CrossFit injuries in Libya, addressing this geographical gap. This study aimed to determine the prevalence, characteristics, and associated risk factors of musculoskeletal injuries among CrossFit athletes in Tripoli, Libya. Study Design: This descriptive, cross-sectional study utilized a convenience sample of CrossFit athletes. Data were collected via a self-administered, paper-based questionnaire adapted from validated epidemiological surveys. Methods: A total of 137 male CrossFit athletes from four affiliated gyms in Tripoli were enrolled. Participants completed a self-administered questionnaire collecting sociodemographic data, training characteristics, and injury history based on a time-loss definition (missing ≥1 training day or seeking medical attention) over a 12-month recall period. Data were analyzed using descriptive statistics. Logistic regression was used to identify injury predictors. Results: The injury prevalence was 40.6%. The shoulder (33.3%) and lumbar spine (25.3%) were the most frequently injured anatomical locations. The primary mechanism of injury was sudden movement (38.6%), and the most common type of injury was tendinopathy (34.5%). The cohort was characterized by relatively young athletes with high training frequency, nearly half of whom had less than six months of training experience. Longer training duration was the only significant independent predictor of injury (OR = 0.136, 95% CI [0.034–0.543] for beginners vs. experienced athletes; *p* = 0.009), indicating that experienced athletes were at higher risk. Conclusions: Libyan CrossFit athletes experience high injury rates, with longer training duration—not novice status—predicting injury. These findings underscore the urgent need for standardized coaching and gym affiliation in developing fitness markets to mitigate technique-related injuries and ensure safe sport participation.

## 1. Introduction

CrossFit is a high-intensity training program that is designed to develop fitness across ten physical parameters: cardiovascular/respiratory endurance, stamina, strength, flexibility, power, speed, coordination, agility, balance, and accuracy [1,2]. It was founded by Greg Glassman in 2000 and had rapid growth. It started as a single gym in California and has grown to over 15,000 affiliated gyms in more than 150 countries these days [3,4].

The CrossFit session follows the “Workout of the Day” (WOD) design, which consists of varied, high-intensity, functional exercises [1]. These workouts combine different exercise forms from Olympic weightlifting (e.g., snatches, clean and jerks), gymnastics (e.g., handstands, ring exercises), and endurance training (e.g., rowing, running) [2,5]. This training approach is designed to mimic the daily activities and tasks that lead to improvements in overall health and fitness, including body composition, cardiorespiratory fitness, and muscular endurance [5,6]. However, the same physiological demands that drive these adaptations also create specific injury mechanisms. Olympic lifts require rapid force production under load, exposing the shoulder complex to high eccentric and compressive forces during overhead movements [7,8]. Simultaneously, the repetitive flexion and extension of the lumbar spine during squats and deadlifts subjects the posterior chain to substantial shear loads, particularly when form deteriorates with fatigue [2,9]. This fatigue-induced breakdown in movement precision is exacerbated by the high-intensity interval format, which compromises neuromuscular control and increases the likelihood of technical errors during complex lifts [5,9]. The CrossFit athletes’ psychological well-being and motivation are attributed to the group support and friendly competitive style of the workouts, which are considered key factors in the popularity and adherence to this sport [10]. The safety of this sport and the risk of injury remain a subject of scientific and professional debate. The nature of the CrossFit sport, which combines high-intensity, complex technical lifts with minimal rest periods, could increase the risk of musculoskeletal injuries, regardless of the documented benefits of CrossFit [7,11]. In addition, difficult and complex techniques are used in this sport; for example, the technical complexity of Olympic lifts, which require near-perfect form to be executed safely, especially when performed for high repetitions by beginners [9].

The existing research on CrossFit injuries is mostly based on cross-sectional surveys. Many studies reported a wide range of injury prevalence, from 20% to over 50% among CrossFit athletes, with incidence rates reaching up to 3.24 injuries per 1000 h of training in different countries [2,7,9,12,13,14]. The shoulder, lower back, and knee were the most frequently injured body parts that were reported consistently across different countries. Regarding the type of injuries sustained in CrossFit sport, the muscle strains and overload injuries sustained during specific exercises like the squat, deadlift, and Olympic weightlifting movements were the most common types of injuries [2,7,8,9].

Most existing research has been conducted in North America, South America, and Europe, where CrossFit gyms are typically affiliated and coaches hold recognized certification. Recent studies continue to report similar injury patterns, with shoulder and lower back injuries predominating and prevalence rates ranging from 28.6% in Germany [15] to 40.6% in the Czech Republic [16], further confirming the consistency of injury profiles across established CrossFit markets [17,18].

In contrast, Libya is considered an emerging and growing CrossFit market that lacks regulatory infrastructure. Gyms run and operate without affiliation and coaches lack certification for this sport. These contextual differences may lead to different and distinct injury patterns not mentioned in the existing literature. In particular, “sudden movement”—a mechanism frequently reported in injury studies—may be especially prevalent in non-affiliated environments, where inadequate coaching oversight allows technical errors to persist and accumulate. Without qualified correction, athletes may attempt complex lifts with poor form, making them vulnerable to acute injuries during high-intensity efforts [15,16].

Despite the rapid growth of CrossFit across Africa, particularly in North African countries like Libya, there is a lack of empirical data and research on this newly introduced and understudied sport. Further, it remains unknown whether injury patterns in non-affiliated settings differ from those of well-established settings. Therefore, this cross-sectional study aims to fill this geographical and scientific gap by examining the prevalence, characteristics (including location and type), and associated risk factors of musculoskeletal injuries among CrossFit athletes in Tripoli, Libya, aiming to provide essential baseline data for the growing CrossFit community in Libya.

## 2. Materials and Methods

### 2.1. Study Design and Setting

Data for this descriptive, cross-sectional study were collected from four CrossFit gyms in Tripoli, Libya.

### 2.2. Participants

Convenience sampling of male CrossFit athletes was used to recruit those aged 14 years or older and who were actively training at one of the participating gyms within Tripoli city. Female athletes were excluded due to their very limited participation in this sport, which results in a very small and inaccessible population. Initially, 262 individuals were invited to participate. Of these, 150 athletes agreed to take part. However, seven of these participants were excluded from the analysis because their returned questionnaires had substantial missing information. Analysis was performed on 143 participants who returned completed questionnaires, yielding a response rate of 54.6% of the initially invited athletes, and 95.3% of those who agreed to participate.

### 2.3. Data Collection Instrument

Data were collected using a paper-based, self-administered questionnaire that was adapted from a previously validated instrument used in CrossFit injury epidemiology research [2,7]. The original English version of the questionnaire was translated into Arabic (the final language of questionnaire administration) to ensure the participants’ understanding, as all participants were fluent in Arabic. The translation process followed standard procedures, including forward translation, back-translation, and pilot testing with 10 Libyan CrossFit athletes to ensure clarity and comprehension. The questionnaire consisted of two main sections. The first section collected demographic and training characteristics, including age, weight, height, education, training frequency, session duration, and years of CrossFit participation. Secondly, injury history was collected, based on a 12-month recall and a standardized time-loss injury definition adapted from previous studies [2,7]. For those who answered yes and reported an injury, additional questions were asked, and more details were collected on the injury characteristics, including the location, type of injury, mechanism of injury, and consequences. A final question was asked to report their participation in other sports. Content validity was ensured through adaptation of validated instruments, and face validity was confirmed during pilot testing. No formal reliability testing was conducted, which we acknowledge as a limitation.

### 2.4. Procedure

Arranged visits to the four participating gyms during September 2025, where a briefing on the study’s objective was given to the gym owners and coaches. A cover sheet was handed to potential participants explaining the study’s purpose. After obtaining informed consent, printed questionnaires were distributed to participants who agreed to participate. The participating minors (ages 14–17) were asked to provide informed consent and permission from their parents. Participants completed the questionnaires on-site and returned them directly to the researchers.

### 2.5. Ethical Considerations

The ethical approval of this study was obtained from the Bioethics Committee at the Biotechnology Research Center (BEC-BTRC) (NBC: 001.H.25.33), confirming that the research complied with ethical guidelines and standards for studies involving human participants.

### 2.6. Data Analysis

Data analyses were conducted using the Statistical Package for the Social Sciences (SPSS), Version 23.0 (IBM Corp., Armonk, NY, USA). Variables were calculated using frequencies and percentages for categorical data, and means and standard deviations for continuous data. The prevalence of injury was measured as the percentage of participants reporting at least one injury in the past year.

In addition, to identify risk factors for injury, inferential analyses were performed. A series of Chi-Square tests was executed to assess the bivariate associations between injury status and key categorical independent variables (duration of training, level of CrossFit participation, level of education, warming-up habits, involvement in other sports, age, BMI, and training volume).

Moreover, a multivariate binary logistic regression analysis was performed to identify the independent predictors of injury while controlling for potential confounders. All variables assessed in the univariate analysis were included using the Enter method to construct the multivariate binary logistic regression model. All results in the multivariate binary regression analysis are presented as odds ratios (OR) with 95% confidence intervals (CI). The statistical significance was set at *p* < 0.05 for all analyses.

## 3. Results

### 3.1. Participants’ Characteristics

The participating CrossFit athletes in this study were relatively young adults with a mean age of 26.5 years. The majority of participants reported a high training frequency, with 81.1% training 5 days per week, and typically for one-hour sessions (91.6%). Almost half of the participants were relatively new to the discipline and participated in other sports as well, with 49.0% practicing CrossFit for less than six months and 49.7% reporting participating in other sports, with football being the most common. Further, 58 out of 143 participants (40.6%) mentioned having experienced some injury while practicing CrossFit. All details on participants’ demographics and training characteristics are illustrated in Table 1.

### 3.2. Injury Characteristics

The shoulder joint and lumbar spine were the most common sites of injury (33.3% and 25.3%, respectively). Further, sudden movement was reported as the primary mechanism of injury (38.6%), followed by heavy weightlifting (28.6%). Furthermore, tendinopathy and other conditions, such as muscle strains and spasms, were the most frequently reported types 34.5%, 36.2%, respectively. Details of the injuries’ characteristics are illustrated in Table 2.

### 3.3. Risk Factors for Injury

Significant associations were found between injury status and both duration of training (*p* < 0.001) and level of CrossFit participation (*p* < 0.001), as shown in the univariate analysis presented in Table 3. The duration of training was the only independent and significant predictor of injury (*p* = 0.009), as estimated by the multivariate logistic regression model, controlling for age, BMI, training volume, and other sports participation. The athletes in the shortest training duration category had significantly lower odds of injury compared to those in the longest duration category (OR = 0.136, 95% CI [0.034, 0.543]). This indicates that beginner athletes were at less risk of getting injured than their experienced counterparts. Details of the multivariate logistic regression model analysis are shown in detail in Table 4.

## 4. Discussion

To the best of our knowledge, this is the first study to investigate the injury prevalence and patterns of injuries among CrossFit athletes in Libya, and also compares them to results reported in different countries. A significant number of Libyan CrossFit athletes experienced injuries (40.6%). The injury prevalence rate among Libyan CrossFit athletes exceeds the reported injury rates in Brazil (31%) and the United States (32.5%) [9,12], and is higher than recently reported rates in Germany (28.6%) [15] and the Czech Republic [16]. Our findings contradict the initial assumption and trends in the existing literature that link the risk of injuries with novice status [9]. Our results revealed a more complex and unexpected risk profile. The most significant independent predictor of injury was a long training duration, as identified in the logistic regression model of this study. Specifically, the most experienced athletes (e.g., those training for more than two years) had significantly higher odds of injury compared to beginners. This finding aligns with recent work by Ferreira et al., who identified years of CrossFit training as a significant factor associated with injury among Portuguese athletes [17]. Similarly, Naderi et al. reported that prior injury history strongly predicted future injuries, suggesting that accumulated training years may confer risk through repeated tissue stress or unresolved previous injuries [19]. In addition, research from Brazil revealed that competitive athletes demonstrated similar injury patterns [20].

In the Libyan context, this increased risk among experienced athletes may be further compounded by structural factors. The lack of coaching standardization and experience could be attributed to many factors, namely, none of the gyms in Libya were officially CrossFit-affiliated, and not all the coaches in these gyms were officially CrossFit-affiliated coaches. Without qualified oversight, technical errors may persist and accumulate over time, and prior injuries may be inadequately rehabilitated, leaving experienced athletes vulnerable despite, or perhaps because of, their longer training history.

Researchers hypothesize that prolonged training durations may result in a cumulative risk of injury. Further, a lack of consistent, high-quality affiliated coaching may allow technical errors to happen, which leads athletes to develop poor movement patterns. Furthermore, these technical deficiencies and errors may become intensified as they progress to higher intensities over months and years, which may lead to the observed higher injury rate among experienced participants. However, these findings are derived from a sample drawn exclusively from gyms in Tripoli, which may not represent the experiences of CrossFit athletes in other Libyan cities or rural areas where access to facilities, coaching, and training culture may differ. Future research should explore whether similar injury patterns exist across broader geographic regions within Libya before these results can be generalized nationally. Therefore, the focus should shift from the high risk to beginners stated in the literature to a critical need for standardization of coaches and gyms. This is achieved by universal affiliation programs for coaches and gyms in developing CrossFit markets.

The results of this study revealed that the shoulder and lower back were the most common sites of injuries; this consistently aligns with CrossFit studies worldwide, from Brazil to the Netherlands [2,13,21,22], and is further supported by recent epidemiological work in the Czech Republic [16] and a systematic review by Bruno et al. [18]. The distribution of these injuries contributes to the technical nature of the CrossFit movements, as the shoulder joint is subjected to high-repetition overhead lifts like snatches and muscle-ups, while the lower back is subjected to substantial loading during deadlifts and squats, especially when form breaks down due to fatigue [7,8]. The consistency of these findings confirms the inherent vulnerability to the demands of the sport, regardless of the practice location.

However, the most significant finding concerning the cause of injury was that participants of this study reported a “sudden movement” as the cause (38.6%), which contradicts the “overuse” narrative often seen in more established CrossFit communities [2,13]. Interestingly, recent research suggests that injury mechanisms may vary by training context. Lenz et al. found that German athletes most frequently attributed injuries to specific exercises like Olympic lifts [15], while Schlegel et al. reported that Olympic lifts and hanging movements were particularly risky in Czech athletes [16]. Although this contradicts findings in some of the existing literature, we hypothesize that this reflects a challenge specific to athletes in developing CrossFit markets like Libya, where coaching standardization and technical foundation may be lacking. For athletes still mastering complex lifts, a “sudden movement” often signals a technical failure, a loss of control under a heavy load, or a loss of control during a fast-paced workout. This often results from pushing intensity without a solid movement foundation to support it [9]. This idea is supported by the most common type of injury reported: tendinopathy (34.5%). Even though tendinopathies are commonly associated with gradual overuse, they could suddenly be aggravated by a single incident of poor mechanics or an unexpected strain [9].

A clear profile of the Libyan CrossFit scene can be established by integrating these findings. The large proportion of participants in this study are committed, enthusiastic, and beginner athletes who regularly train, but the high injury rate and the specific mechanism of “sudden movement” injuries suggest a gap between their enthusiasm and their technical proficiency. In addition, the competitive nature of CrossFit sport, where the intense, competitive aspects undermine the essential, careful building of movement skills [5]. Thus, a lack of strong emphasis on foundational technique and qualified and affiliated coaches will leave the athletes more vulnerable to injury.

This study provides initial descriptive data on CrossFit injuries in Libya, which may inform future investigations in this setting. However, this study has several limitations. Firstly, the cross-sectional design of the study does not prove the cause and effect. While this design was appropriate for an initial investigation in a previously unstudied population, future research should employ prospective cohort designs to better understand temporal patterns and causal mechanisms. Secondly, data were collected only from male athletes due to the absence of female CrossFit participants in Libya at the time of the study, which limits the applicability of the findings to female athletes. Thirdly, data were collected from one city (Tripoli), which may limit the generalizability of the findings, as it does not reflect findings from other parts of Libya. Future multicenter studies across different regions of Libya are needed to validate and expand upon these results. Finally, the inclusion of participants aged 14 years and older, while reflective of the actual training population in Libyan CrossFit gyms, may introduce age-related heterogeneity. However, given the mean age of 26.5 years and the small proportion of minors in the sample, this is unlikely to have substantially affected the findings. Additionally, this study did not assess potential confounding factors such as body composition, pre-existing medical conditions, dietary habits, biomechanical variables, or training and rest periodization, all of which may influence injury risk and should be considered in future research.

### Clinical Implications

Gym affiliation and formal education for coaches are mandatory to reduce and prevent injuries associated with CrossFit, especially sudden movements and technical errors. Further, Libyan CrossFit gyms must prioritize safety by mandating foundational technique courses for beginners, providing continuous coaching for experienced athletes to correct poor form, and integrating specific prehabilitation for the vulnerable shoulder and spine.

## 5. Conclusions

Libyan CrossFit athletes experience a 40.6% injury prevalence, predominantly affecting the shoulder and lumbar spine. Sudden movement was the most commonly reported mechanism, often occurring in the context of fatigue or inadequate recovery, suggesting that both acute events and underlying physiological stress contribute to injury risk. In contrast to some existing literature, longer training duration—not novice status—was the most significant predictor of injury, a finding that may reflect the absence of certified coaching and standardized gym affiliation in this setting. These results underscore the urgent need for coach certification, foundational technique programs for beginners, continued skill development for experienced athletes, and injury surveillance systems in emerging CrossFit markets to protect participants and support sustainable growth.

## Figures and Tables

**Table 1:** Demographic and Training Characteristics of Study Participants.

Variable	Total Sample (*n* = 143)
Age (years), mean ± SD	26.5 ± 8.3
Range (min–max)	15–57
Weight (kg), mean ± SD	83.4 ± 16.4
Range (min–max)	45–140
Height (cm), mean ± SD	175.5 ± 6.7
Range (min–max)	158–191
Level of education	*n* (%)
Basic	55 (38.5)
College	78 (54.5)
Postgraduate	10 (7.0)
Time participating in CrossFit	*n* (%)
<6 Months	70 (49.0)
6–12 Months	27 (18.8)
1–2 Years	25 (17.5)
>2 Years	21 (14.7)
Training days/week	*n* (%)
3 days	8 (5.6)
4 days	11 (7.7)
5 days	116 (81.1)
6 days	8 (5.6)
Training hours/day	*n* (%)
1 h	131 (91.6)
2 h	8 (5.6)
3 h	4 (2.8)
Practice for other sports	*n* (%)
Yes	71 (49.7)
No	72 (50.3)
Other sports	*n* (%)
Football	48 (33.6)
Basketball	5 (3.5)
Running	4 (2.8)
Walking	2 (1.4)
Other	12 (8.4)
Injury prevalence in the last 12 months	58 (40.6)

**Table 2:** Injury characteristics.

Variable	*n* (%)
**Number of Injuries**	
One injury	39 (67.2)
Two injuries	16 (27.5)
Three injuries	2 (3.4)
Four injuries	1 (1.7)
**Site of injury**	
Pelvis	4 (5.3)
Knee	9 (12)
Ankle	2 (2.6%)
Shoulder	25 (33.3%)
Lower back	19 (25.3%)
Neck	3 (4)
Hands	1 (1.3%)
Arm	4 (5.3)
Leg	6 (8)
Elbow	2 (2.6)
**Cause of injury**	
Heavy weightlifting	20 (28.6%)
Repetitions	8 (11.4%)
Sudden movement	27 (38.6%)
Fatigue	7 (10%)
Other	8 (11.4%)
**Type of injury**	
Muscular Stretch	4 (6.9)
Sprain	5 (8.6)
Contusion	6 (10.3)
Fracture	2 (3.4)
Tendinopathy	20 (34.5)
Other (spasm, strain, unknown)	21 (36.2)

**Table 3:** Bivariate Associations Between Participant Characteristics and Injury Status (Chi-Square Test *p*-values).

Variable Tested	*p*-Value
Duration of training	<0.001
Level of CrossFit participation	<0.001
Level of Education	0.136
Warming up	0.188
Participating in other sports	0.682
Age	0.681
BMI	0.435
Training volume	0.521

**Table 4:** Multivariate Logistic Regression Analysis of Factors Associated with Injury Among CrossFit Athletes.

Variable	B	Wald	*p-*Value	OR (Exp(B))	95% CI for EXP(B)	
					Lower	Upper
Age	−0.025	1.062	0.303	0.975	0.929	1.023
BMI	−0.038	0.816	0.366	0.963	0.888	1.045
Level of CrossFit Professional (reference)		4.224	0.121			
Beginner	−1.687	3.093	0.079	0.185	0.028	1.213
Middle	−0.808	0.894	0.344	0.446	0.084	2.379
Duration of CrossFit training More than 2 years (reference)		11.566	0.009			
Less than 6 months	−1.998	7.972	0.005	0.136	0.034	0.543
6 months to 1 year	−1.320	3.293	0.070	0.267	0.064	1.111
1 year to 2 years	−0.351	0.235	0.628	0.704	0.171	2.905
Warming up Sometimes (reference)		2.912	0.233			
Yes	−1.419	2.912	0.088	0.242	0.047	1.235
No	−22.114	0.000	1.000	0.000	0.000	
Participation in other sports	0.014	0.018	0.895	1.014	0.829	1.239
Training volume	−0.012	1.535	0.215	0.880	0.718	1.077

## Data Availability

The data presented in this study are available on request from the corresponding author. The data are not publicly available due to privacy or ethical restrictions.
